# When Crohn’s Disease Is Confined to the Mouth: A Diagnostic Challenge—A Case Report and Review of the Literature

**DOI:** 10.3390/jcm15010004

**Published:** 2025-12-19

**Authors:** Axel Vattier, Justine Chapuis, Marie Orliaguet, Amelie Bourhis, Franck Cholet, Laurent Misery, Sylvie Boisramé

**Affiliations:** 1 Department of Dentistry, Oral Medicine and Oral Surgery, UFR Odontologie, Brest University Hospital, 29200 Brest, France; axel.vattier@etudiant.univ-brest.fr (A.V.);; 2 Laboratoire Interaction Epithelium Neurones, LIEN (UR 4685), Department of Dentistry, Oral Medicine and Oral Surgery, UFR Odontologie, Brest University Hospital, University of Brest, 29200 Brest, France; marie.orliaguet@univ-brest.fr; 3 Department of Pathology, Brest University Hospital, 29200 Brest, France; 4 Department of Gastroenterology, Brest University Hospital, 29200 Brest, France; 5 Laboratoire Interaction Epithelium Neurones, LIEN (UR 4685), Department of Dermatology, UFR Medecine, Brest University Hospital, University of Brest, 29200 Brest, France; 6 Inserm, EFS, UMR1078, GGB, Department of Dentistry, Oral Medicine and Oral Surgery, UFR Odontologie, Brest University Hospital, France University of Brest, 29200 Brest, France

**Keywords:** Crohn’s disease, oral, biotherapies, gingivitis, oro-facial granulomatosis

## Abstract

This article explores a rare and diagnostically challenging form of Crohn’s disease, known as Oral Crohn’s Disease (OCD), in which the condition is confined to the oral cavity without gastrointestinal involvement. Additionally, Crohn’s disease is typically associated with digestive manifestations, and oral lesions may occasionally represent the first- or even the sole- signs of the disease, making diagnosis difficult due to their non-specific presentation. We report the case of a 22-year-old presenting woman suffering from chronic painful gingivitis and macrocheilitis, in the absence of gastrointestinal symptoms. Despite multiple topical treatments and an initial non-specific histopathological report, a multidisciplinary case discussion and re-evaluation of biopsies led to the diagnosis of OCD. Comprehensive gastrointestinal assessments revealed no intestinal involvement. Owing to the persistence of symptoms and resistance to topical therapies, the patient was subsequently treated with an anti–TNFα (Tumor Necrosis Factor alpha) biologic agent. To contextualize this case, we conducted a literature review and identified six similar cases published between 2000 and 2025. Reported patients presenting with symptoms such as lip swelling, cheilitis, mucosal ulcerations, and gingivitis. Histopathological findings consistently demonstrate non-caseating granulomas and inflammatory cell infiltration. Most cases responded favorably to corticosteroids, while some required systemic or biologic therapy. The article highlights that OCD remains underrecognized due to its variable clinical presentation and absence of gastrointestinal manifestations. It emphasizes the importance of integrating clinical, histological, and exclusion-based diagnostic criteria and advocates for a multidisciplinary approach involving dental surgeons, dermatologists, pathologists, and gastroenterologists. Early recognition and long-term monitoring are essential, as gastrointestinal involvement may develop years after the onset of oral symptoms.

## 1. Introduction

Crohn’s disease is a chronic inflammatory bowel disease that can affect the entire digestive tract, from the mouth to the anus [1]. It is characterized in the majority of cases by digestive involvement with marked symptomatology such as diffuse abdominal pain, recurrent diarrhea, or even rectal bleeding [2].

The etiopathogenesis of this disease remains unknown to this day, although multiple factors seem to be involved, such as immunological, environmental, microbiota, and genetic factors. Dermatological manifestations are present in 22 to 44% of patients with Crohn’s disease [3]. According to Veiga et al. [1], in 60% of cases, the oral involvement may be the first sign. We know today that the oral manifestations of the disease can coincide with or precede the digestive involvement by several years. These oral manifestations are multiple and not pathognomonic of the disease. They include, in particular, facial edema, mucosal ulcerations, mucosal hypertrophy (cobblestone-like or paved aspect, inflammatory gingivitis).

The management of these oral lesions in first intention is based on topical corticosteroid treatment. In cases of total or partial failure, or when a digestive involvement of the disease is discovered, systemic corticosteroid therapy or biotherapy such as anti–Tumor Necrosis Factor (TNF)α may be implemented.

Despite increasing recognition of Crohn’s disease, isolated oral manifestations remain poorly characterized in the literature, leading to diagnostic delays and therapeutic uncertainty. This study aimed to identify the diagnostic and therapeutic criteria for oral Crohn’s Disease (OCD) by reviewing the literature and to present a clinical case that illustrates the diagnostic challenges associated with this condition.

## 2. Materials and Methods

### 2.1. Illustrative Case

A 22-year-old woman, an accounting assistant, was referred by her general dental surgeon to the specialized oral dermatology consultation of the University Hospital Center of Brest, France, in July 2022 for a painful gingivitis evolving for several years with a pattern of painful flares and remission phases. Notable medical history in the patient included growth retardation treated with growth hormones, a Haglund’s disease of the right ankle treated by calcaneum resection, early puberty, and an anal fissure. It was also noted the presence of fibromyalgia and a history of breast cancer in the patient’s mother.

**Figure 1:**
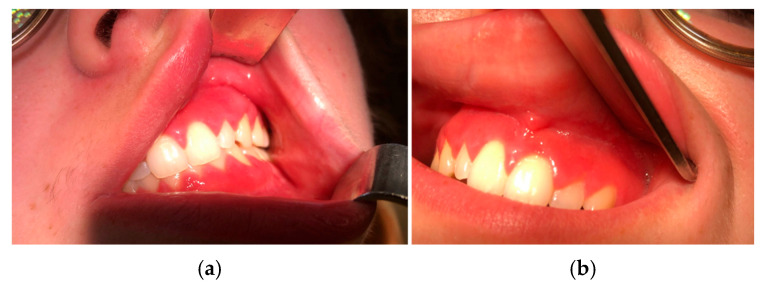
(**a**,**b**): Initial macroscopic aspect of inflammatory gingivitis.

Clinical examination revealed inflammatory gingivitis affecting the entire height of the keratinized gingiva on both dental arches (Figure 1a,b). The rest of the clinical examination was unremarkable.

As an initial approach, a diagnosis of plasmacytic gingivitis was considered, and topical corticosteroid therapy (prednisolone mouth rinse 20 mg three times per day) was initiated. Two biopsies were also performed and sent to the laboratory for anatomopathological examination.

**Figure 2:**
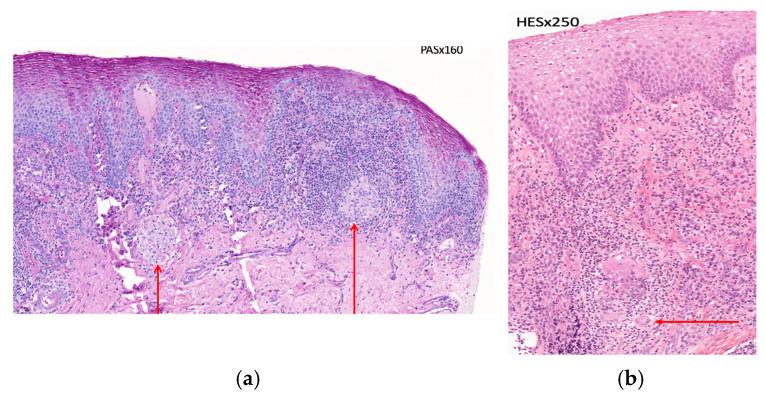
(**a**). Histological sections of gingival biopsy: —Granuloma (arrows)—PAS staining, ×160. (**b**). Histological section of gingival biopsy: —Giant cell (arrow)—HES Staining × 250.

Histological analysis revealed an acanthotic squamous epithelium, without hypergranulosis, exhibiting spongiosis and exocytosis of mononuclear inflammatory cells. The basement membrane showed focal vacuolization and was partially obscured by the underlying inflammation, with no evidence of apoptosis. The lamina propria demonstrated a dense interstitial mononuclear infiltrate, rich in plasma cells, along with some granulomatous formations containing multinucleated giant cells (Figure 2a,b). Special stains did not reveal any fungal or parasitic pathogens, and no immunostaining with anti-treponemal antibody was detected. These findings were consistent with nonspecific plasmacytic gingivitis.

**Figure 3:**
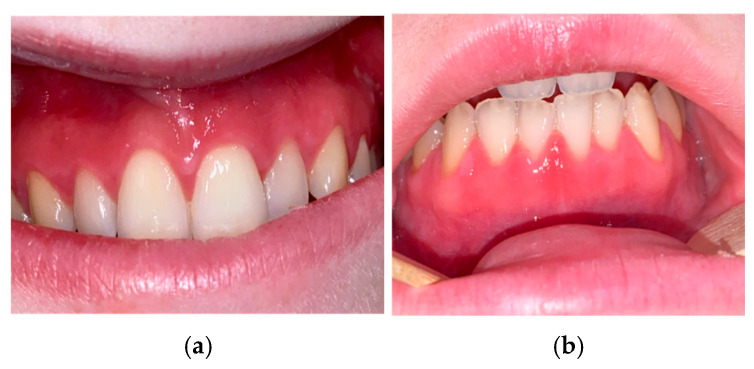
(**a**,**b**): Aspect of the inflammatory gingivitis (February 2023).

One month later, no clinical improvement was observed. The topical corticosteroid therapy was therefore changed to betamethasone mouth rinses at the same dosage. In addition, topical tacrolimus was prescribed twice a day as a subsequent therapeutic option should the condition fail to improve. During the period from August 2022 to February 2023, despite the implementation of multiple topical treatments (prednisolone, tacrolimus, clobetasol propionate, betamethasone dipropionate), no improvement was observed in the inflammatory gingivitis (Figure 3a,b).

**Figure 4:**
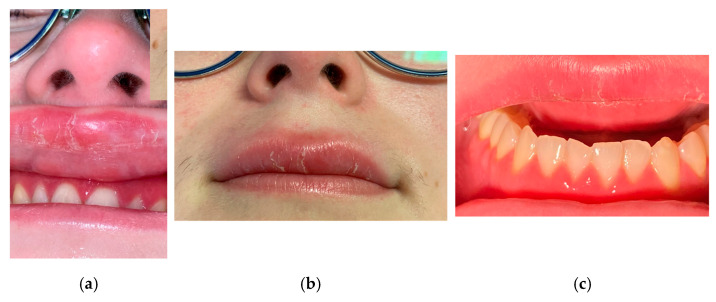
(**a**,**b**). Macroscopic aspect of upper labial swelling (December 2024). (**c**). Intra-buccal photography of attached gingiva (December 2024).

The appearance of an upper fissured macrocheilitis was noted, in addition to the gingivitis, further aggravating the patient’s concerns regarding the esthetics of her face and smile (Figure 4a–c).

The patient’s medical history and the clinical evolution prompted consideration of differential diagnoses, including Crohn’s disease and orofacial granulomatosis (OFG). A blood test with fecal calprotectin assay was carried out, along with a comprehensive assessment for OFG including an electrolyte panel, creatinine, albumin, serum calcium, CRP (C-reactive protein), liver function tests, thyroid-stimulating hormone, testosterone, cortisol, adrenocorticotropic hormone, serum proteins, angiotensin-converting enzyme, and a complete blood count; however, no significant abnormalities were identified.

In January 2025, the case was presented at a multidisciplinary discussion meeting of the GEMUB (Groupe d’Etude de la Muqueuse Buccale—French Oral Mucosa Study Group), which brings together several medical specialties (dermatologists, oral surgeons, maxillofacial surgeons, and ear, nose, and throat specialists, …) involved in the study of orofacial diseases. The discussion concluded with a suspicion of OCD. Consequently, a request was made for the histopathological slides to be reread and for a consultation with a gastroenterologist to investigate the potential for gastrointestinal involvement. The histopathological review confirmed the presence of giant-cell granulomas, with an epithelioid component and without caseous necrosis. These granulomatous changes, although not entirely specific, were consistent with Crohn’s disease in the given clinical context.

Gastroenterological evaluations, including intestinal-MRI and small bowel video capsule endoscopy, standard upper gastrointestinal endoscopy, and ileocolonoscopy, revealed no luminal involvement. Furthermore, the anal fissure observed was considered atypical for Crohn’s disease. The diagnosis of OCD was therefore established, based on the clinical findings, histopathological evidence, and the exclusion of differential diagnoses.

**Figure 5:**
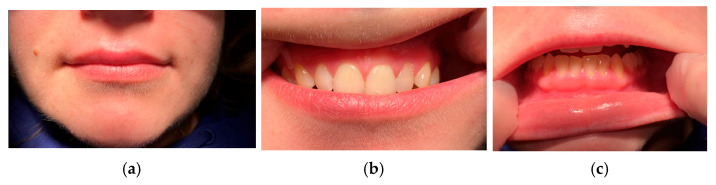
(**a**). Macroscopic aspect after treatment. (**b**,**c**). Intra-buccal photography of attached gingiva (December 2025).

Given the limited efficacy of topical therapies, biologic treatment with an anti-TNF α agent (Adalimumab) was initiated at a loading dose of 80 mg, followed by 40 mg every two weeks, after completing a pre-therapeutic screening to rule out infectious contraindications (dental examination, chest radiography, blood test including hematological, biochemical, immunological, and virological laboratory investigations). The patient was seen again for a follow-up consultation recently, and due to the presence of a treatment-related reactive tachycardia that was affecting her daily life, the therapy was switched to another anti-TNFα agent, certolizumab. Since the initiation of the treatment, the patient has no longer presented any symptoms, and both the extraoral and intraoral clinical examinations demonstrate an excellent therapeutic response with regression of the lesions (Figure 5a–c). In order to better understand and contextualize this case report, observations with cases and data reported in the international literature were compared.

### 2.2. Search Strategy

A computerized literature search was performed in the PubMed, Web of Science, and Scopus databases using the following search equation: “Oral AND Crohn’s disease.”

The search was limited to articles in English or French since 2000.

### 2.3. Selection Criteria

The inclusion criteria were case reports written in French or English between 2000 and 2025, as well as the presence of oral symptomatology only. The exclusion criteria were systematic reviews and articles presenting case reports with gastrointestinal symptoms of the disease. The titles were screened for relevance, and duplicates were removed.

### 2.4. Data Extraction

The following data were extracted after a full-text review of all selected articles, authors, country, population characteristics, symptoms, examinations, histopathological features, treatment, and follow-up period.

## 3. Results

**Figure 6:**
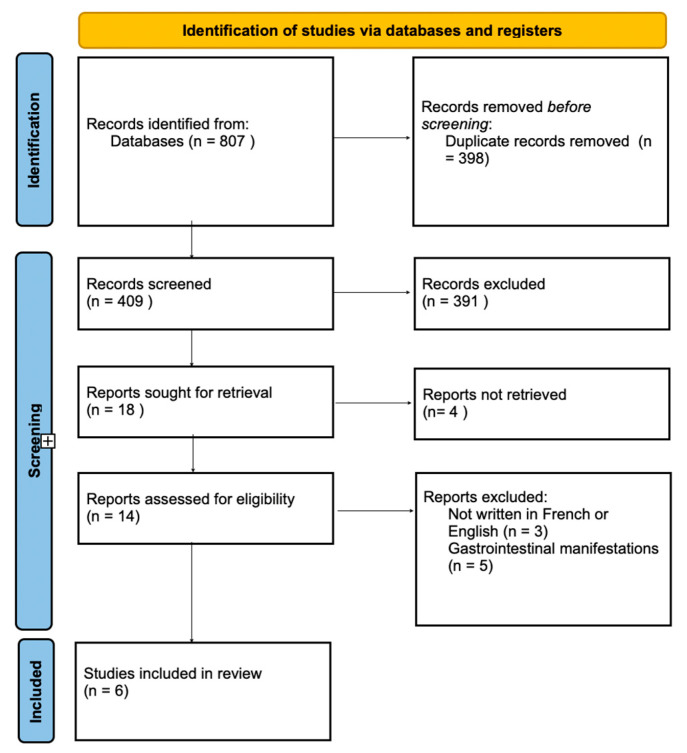
Flow diagram of the process for narrative review.

The study search process is summarized in the flow diagram (Figure 6).

The initial search found a total of 807 articles. After duplicates were removed and the remaining articles were screened, a total of six clinical cases were identified and included for data extraction. The majority were excluded after title and abstract screening because they did not report isolated OCD.

Table 1 summarizes the results of the literature review and the characteristics of included studies. The affected patients were predominantly male (five men and one woman), with ages ranging from 10 to 64 years (mean age: 34 years). The follow-up period varied from a few weeks to approximately 18 months. Oral clinical manifestations reported included labial edema [1,4,5,6], cheilitis [4,6,7], oral ulcerations [1,7,8], gingivitis and erythema [1,4], and a cobblestone-like (pavement-like) appearance of the mucosa [1,8]. No notable medical history was reported among the patients included in these cases.

In all studies, intraoral biopsies were performed for histopathological examination. These examinations revealed, in five of the reviewed cases, the presence of giant-cell granulomas without caseous necrosis. An intense lymphocytic infiltration associated with other immune cells, particularly macrophages—indicative of marked inflammation of the underlying connective tissue—was also observed in all cases.

Additionally, complementary examinations included blood tests in all cases (CRP and fecal calprotectin), chest radiographs in three cases, and gastroenterological examinations aimed at identifying potential gastrointestinal involvement. These examinations comprised endoscopy [7], video capsule endoscopy [1], colonoscopy in three cases, and intestinal biopsies for histopathological assessment [5,6,7].

Therapeutic strategies were initially based on oral corticosteroid therapy [5,6,7] and/or topical corticosteroids, either in mouthwash form [1] or as topical cream form [8]. Three authors employed oral corticosteroids as first-line therapy [5,6,7]. In one case [1], anti-TNF α biotherapy (Infliximab) was introduced following the diagnosis of OCD. Overall, the clinical evolution was favorable in all reported cases, with few complications and clear improvement in both the clinical appearance of the oral lesions and the patients’ symptomatology.

## 4. Discussion

The results of this study highlight the diagnostic challenges of OCD, which remains likely underdiagnosed to this day. The analysis of reported cases demonstrates that the oral manifestations of OCD are highly heterogeneous and often non-specific. The most commonly observed lesions included labial swelling, angular cheilitis, and mucosal ulcerations, sometimes associated with inflammatory gingivitis or a cobblestone-like (pavement-like) appearance of the mucosa. These features, when persistent and resistant to conventional therapies, should raise suspicion for OCD. However, diagnosis is often complicated by clinical overlaps with other granulomatous or inflammatory conditions such as sarcoidosis, OFG, and chronic infections. Histopathological findings- typically non-caseating granulomas can support the diagnosis but are not pathognomonic and may be absent in early or mild lesions.

Several reports [6,8] noted the absence of gastrointestinal involvement despite extensive complementary investigations. This finding underscores that the oral manifestation of Crohn’s Disease (OCD) may precede intestinal involvement by several years [1].

Histopathological examination remains the gold standard for diagnosis. The presence of non-caseating epithelioid granulomas, associated with lymphoplasmacytic infiltration and multinucleated giant cells, was identified in the majority of cases.

However, these histological features are not pathognomonic and may also be observed in other forms of OFG, such as sarcoidosis or Miescher’s cheilitis [4]. These entities should therefore be considered in the differential diagnosis of this clinical presentation.

The diagnosis of OCD thus relies on a combination of clinical findings, histopathological evidence, and the exclusion of other granulomatous disorders. The management of OCD remains challenging due to its chronic, relapsing nature and variable response to therapy. Treatment strategies aim to reduce inflammation, alleviate symptoms, and prevent recurrence while addressing any associated intestinal disease.

Therapeutic management of mild oral lesions in OCD is primarily based on corticosteroid therapy (such as triamcinolone acetonide or Clobetasol propionate), administered topically as ointments, gels, or mouth rinses. In more extensive or refractory lesions, systemic corticosteroids and immunomodulators such as azathioprine, methotrexate, or six mercaptopurine may be required.

Most reported cases demonstrated significant improvement following topical corticosteroid treatment, although some required systemic relay [5,7]. In refractory or recurrent forms, particularly when gastrointestinal involvement is confirmed, anti-TNFα biotherapies such as infliximab or adalimumab have proven effective [1]. Anti-TNFα antibodies in this type of pathology aim to reduce inflammation by regulating the pro-inflammatory cytokine TNFα, which is involved in the pathogenesis of chronic inflammatory bowel diseases such as Crohn’s disease.

Overall, most cases in the literature reported a favorable outcome with corticosteroids or biologics, though recurrences may occur. Therapy must be individualized according to the extent of oral involvement, functional impairment (e.g., mastication, speech), and psychosocial impact. Supportive care, such as maintaining good oral hygiene, avoiding irritant foods, and correcting nutritional deficiencies, is also essential.

In the clinical case presented, the persistence of inflammatory gingivitis and the subsequent development of macrocheilitis, despite multiple topical treatments, led to the suspicion of a granulomatous disease. The final diagnosis of OCD was established after histopathological reevaluation and exclusion of other granulomatous etiologies. This case exemplifies the diagnostic challenges and potential delays that may extend over several years before a definitive diagnosis is reached. Several reports have documented isolated or extraintestinal localizations without overt intestinal involvement.

Compared with the cases in Table 1, our patient displayed the typical oral features of OCD, in which inflammatory changes led to lip swelling accompanied by granulomatous gingivitis, and biopsy confirmed non-caseating granulomas, as reported in most cases. The insufficient response to topical therapy and the need for systemic treatment also mirror refractory cases. Overall, the presentation fits within known patterns while illustrating the clinical variability of isolated OCD.

The oral cavity represents the most frequently reported site of isolated Crohn’s disease, with some studies estimating its prevalence at approximately 0.5% of all Crohn’s disease cases. However, isolated forms have also been reported in other regions, including the esophagus, stomach, and duodenum, with or without later intestinal extension [9]. Similarly, isolated cutaneous Crohn’s has been described in the absence of gastrointestinal lesions [10]. These observations suggest that Crohn’s disease may, in rare circumstances, remain confined to specific tissues for extended periods, representing a localized immune-mediated granulomatous process rather than systemic intestinal inflammation.

These observations highlight the importance of a multidisciplinary approach in the management of patients presenting with chronic and atypical oral lesions. Collaboration among dental surgeons, oral surgeons, dermatologists, pathologists, general practitioners, and gastroenterologists is essential to minimize diagnostic delay and to ensure the implementation of appropriate therapy.

Because OCD is a rare entity and often underrecognized, early recognition is critical.

The clinical warning signs, which include lip swelling, macrocheilitis, and granulomatous gingivitis, should alert clinicians. Long-term follow-up of patients is also crucial, as gastrointestinal involvement may develop secondarily, sometimes years after the initial onset of oral manifestations.

The main limitation of this review lies in the small number of published cases, consisting mainly of isolated clinical reports with limited follow-up, which preclude the establishment of standardized diagnostic or therapeutic recommendations for this condition.

Studies exploring the immunologic and microbial pathways involved in oral lesion formation could shed light on why some patients develop oral manifestations while others do not. Advances in microbial profiling, salivary biomarkers, and molecular diagnostics may enable earlier and more accurate identification of OCD. Developing standardized clinical and histopathological diagnostic criteria is also essential to distinguish OCD from other granulomatous conditions. On the therapeutic side, evaluation of newer biologic agents, small-molecule inhibitors, and innovative topical delivery systems could provide more effective and targeted treatments. Finally, further longitudinal studies involving larger cohorts and interdisciplinary research integrating gastroenterology and oral medicine are needed to improve understanding of OCD, its natural course, diagnostic criteria, and optimal management strategies.

## 5. Conclusions

The diagnosis of OCD often proves challenging, particularly when gastrointestinal symptoms are minimal or absent. It relies primarily on clinical evaluation, histopathological examination, and the exclusion of differential diagnoses.

Therefore, this diagnosis should be considered in the presence of orofacial granulomatous lesions that are resistant to conventional local treatments. Notably, such oro-facial manifestations may precede or occur in the absence of digestive involvement, thus enabling early recognition of the disease.

Clinicians, especially dentists, oral surgeons, and dermatologists, should carefully distinguish OCD from other granulomatous disorders, such as sarcoidosis, OFG, and infectious etiologies, by combining clinical patterns, investigations, and histology.

This condition requires close multidisciplinary follow-up involving dental surgeons, oral surgeons, and gastroenterologists. Such collaboration is essential to ensure early detection of potential gastrointestinal involvement and to optimize patient outcomes.

While isolated oral cases may follow a relatively indolent course, long-term monitoring is recommended, as some patients may develop a systemic disease over time.

## Figures and Tables

**Table 1:** Results of the study.

References	Country	Age/Sex	Main Oral Symptoms	Key Point Findings	Treatment	Evolution
Thomas Williman et al., 2007 [5]	Germany	10/M	Lip edema with pain	Lymphocytic Infiltrate	Oral prednisolone	Improvement (a few weeks)
Padmavathi BN et al., 2014 [4]	India	34/M	Edema Angular cheilitis Erythema	Non-caseating granulomas	Not specified	Not specified
Gingisetty, Harikishan et al., 2012 [6]	India	22/F	Edema Fissure Angular cheilitis	Multiple Granulomas	Topical steroids Gingivectomy	Significant improvement (15 mo)
Hamid, Salek et al., 2014 [8]	USA	64/M	Ulceration Fissure	Non-caseating granulomas	Topical steroid	Complete healing (9 mo)
Henedina Antunes et al., 2015 [7]	Portugal	17/M	Ulceration Cheilitis	Granulomas	Prednisolone + Azathioprine	Improvement (4 w)
Filipa, Veiga et al., 2023 [1]	Portugal	61/M	Gingivitis Lip edema Ulcers	Granulomas Negative infectious test	Steroids → Infliximab + Methotrexate	No recurrence (17 mo)

## Data Availability

The Data will only be made available from the corresponding author upon reasonable request.

## Abbreviations

OCD	Oral Crohn’s Disease
TNFα	Tumor Necrosis Factor alpha
OFG	Oro Facial Granulomatosis
CRP	C-Reactive Protein
